# Compliance With DVLA Guidelines Within an Early Intervention in Psychosis Service

**DOI:** 10.1192/bjo.2025.10631

**Published:** 2025-06-20

**Authors:** Charlotte Matheson, Rachael Elliott

**Affiliations:** 1 Doncaster and Bassetlaw Teaching Hospitals NHS Foundation Trust, Doncaster, United Kingdom; 2 Sheffield Teaching Hospitals NHS Foundation Trust, Sheffield, United Kingdom; 3 University of Liverpool, Liverpool, United Kingdom

## Abstract

**Aims:** Early Intervention in Psychosis (EIP) services provide specialised support for individuals experiencing their first episode of psychosis. Psychosis can impair cognitive and motor skills, which may affect an individual’s ability to drive safely. If a patient’s fitness to drive may be impaired by their condition or treatment, both DVLA and the GMC advises doctors to (i) alert patients to this issue, (ii) inform patients of their legal obligation to notify the DVLA, and (iii) document advice regarding fitness to drive in the patient’s medical records. This audit aimed to assess the EIP teams’ compliance with these guidelines.

**Methods:** A retrospective audit of 52 patients referred most recently to the EIP services. Online patient case notes were reviewed for information on (i) driving status, (ii) licence status, (iii) information provided on driving, including the need to inform DVLA.

**Results:** 59% (n=31) patients had driving status recorded by the EIP team, 81% (n=42) had licence status recorded. 10% (n=5) had no information about their driving or licence status. 27% (n=14) of patients received general advice about driving by the EIP team.

31% (n=16) of patients held a current driving licence, 69% (n=11) of these received advice about driving, 56% (n=9) were advised to inform the DVLA.

**Conclusion:** The majority of patients with a current driving licence received driving advice. However, a significant proportion did not, posing risks to both patients and the public. The EIP team should aim for 100% compliance with the DVLA/GMC guidance.

Requesting information about driving status is already included in the EIP clerking proforma. To improve adherence to completing this documentation, the audit findings will be presented to the multi-disciplinary team to raise awareness and reinforce the importance of this section. In addition, a poster with proforma screenshots will be distributed to support colleagues in locating it this section. A re-audit in six months will assess progress and determine if further interventions are needed.

